# Preimplantation Genetic Testing: Where We Are Today

**DOI:** 10.3390/ijms21124381

**Published:** 2020-06-19

**Authors:** Ermanno Greco, Katarzyna Litwicka, Maria Giulia Minasi, Elisabetta Cursio, Pier Francesco Greco, Paolo Barillari

**Affiliations:** 1 Reproductive Medicine, Villa Mafalda, 00199 Rome, Italy; ergreco1@virgilio.it (E.G.); mg.minasi@gmail.com (M.G.M.); elicur@hotmail.it (E.C.); piergreco@hotmail.com (P.F.G.); paolo.barillari@villamafalda.com (P.B.); 2 UniCamillus, International Medical University, 00131 Rome, Italy

**Keywords:** preimplantation genetic screening, in-vitro fertilization, biopsy, euploid embryo, implantation, pregnancy, endometrium, mosaicism

## Abstract

Background: Preimplantation genetic testing (PGT) is widely used today in in-vitro fertilization (IVF) centers over the world for selecting euploid embryos for transfer and to improve clinical outcomes in terms of embryo implantation, clinical pregnancy, and live birth rates. Methods: We report the current knowledge concerning these procedures and the results from different clinical indications in which PGT is commonly applied. Results: This paper illustrates different molecular techniques used for this purpose and the clinical significance of the different oocyte and embryo stage (polar bodies, cleavage embryo, and blastocyst) at which it is possible to perform sampling biopsies for PGT. Finally, genetic origin and clinical significance of embryo mosaicism are illustrated. Conclusions: The preimplantation genetic testing is a valid technique to evaluated embryo euploidy and mosaicism before transfer.

## 1. Introduction

IVF is a reproductive technique whose success rate depends on several steps: ovarian stimulation, egg retrieval, embryo culture, and transfer. Embryo implantation is one of the most critical point in every IVF program and transfer of a vital embryo in a receptive endometrium is essential for achieving a pregnancy in an assisted reproduction cycle. Despite many improvements reached today the process of embryo implantation is still very ineffective [1].

Therefore, the selection of the best embryo to transfer is the main challenge to face, mostly when a single embryo transfer (SET) program is adopted for different clinical reasons. As currently practiced, the embryo that is chosen for transfer is selected on morphologic grading criteria, which has significant inter- and intraobserver variability [2]. At the cleavage stage, the number of cells, their symmetry, and the presence of cellular fragments are evaluated. At the blastocyst stage, the evaluated parameters are blastocyst expansion and the inner cell mass and trophoectoderm appearance. Today, there is a wide consensus that the microscopic appearance of an embryo is weakly correlated with its viability [3,4]. Thus, a variety of non-invasive methods, such as time-lapse imaging for embryo morphokinetics [5], proteomic [6], and metabolomic [7] study, was proposed to assess the embryo quality. Extending embryo culture to the blastocyst stage was shown to improve outcomes from SET [8], although morphologically normal blastocysts still retain a significant risk of aneuploidy [9,10,11,12]. Therefore, the clinical outcomes from SET have been demonstrated to be lower in several randomized controlled trials performed to date and confirmed by subsequent meta-analysis [13,14]. The transfer of multiple embryos is frequently utilized in clinical practice to improve the chance of implantation, but this approach increases the risk of multiple pregnancies [15,16].

At the same time, several studies have demonstrated that embryo aneuploidy is the most important reason of IVF failure, enhancing the importance of preimplantation genetic testing for aneuploidies (PGT-A) as a method for selecting chromosomally healthy embryos [17,18,19]. Aneuploidies in human embryos are strictly correlated with female age [20] and are derived from chromosomal errors that can occur at different levels. Meiotic errors occur during oogenesis: the prolonged arrest of oocyte development in prophase results in a degradation of the meiotic apparatus. Mitotic errors happen after fertilization, usually during the first mitotic divisions and lead to embryo mosaicism. Sperm aneuploidies, generally correlated with sperm quality and DNA fragmentation, are less common if compared to oocytes ones, but their incidence in embryo aneuploidy is reported to be high [21].

PGT-A was introduced for the first time in the 1993 to select euploid embryos to transfer and improve assisted reproductive results [22]. However, the first generation PGT was demonstrated to be less effective in improving IVF live birth (LB) rates and reducing miscarriage rates [23] mainly due to the incomplete assessment of chromosomal status and undiagnosed mosaicism deriving from post-zygotic cleavage division errors in day-3 embryo [24]. In fact, in the beginning this screening was performed using Fluorescence in Situ Hybridization (FISH), which analyzed only a reduced number of chromosomes. The need to investigate embryos ploidy status led to the development of different techniques for the analysis of the whole chromosomal panel, such as Array-Comparative Genomic Hybridization (aCGH), Next Generation Sequencing (NGS), and Real Time Quantitative Polymerase Chain Reaction (rtq-PCR). The biopsies for the analysis can be removed from the oocyte, collecting the first and/or second polar body or from the cleavage stage embryo, removing some blastomeres or from the blastocyst, collecting some trophoectoderm cells. These techniques can be applied for different indications in which the transfer of euploid embryo might improve the clinical outcomes.

## 2. PGT Techniques

### 2.1. Polar Body Biopsy

Polar bodies (PB) are by-products of the meiotic divisions of the oocyte. They have no reproductive function and can be easily removed without affecting embryo development. PB biopsy can be applied in a single step or two step method: the first consist in the simultaneous removal of the first and second PB sixteen h after insemination, whereas the two steps method entails two different biopsies, one prior to intracitoplasmatic sperm injection (ICSI), with the removal of the first PB, and the other sixteen h after insemination, with the removal of the second PB. The single step procedure would seem to be more convenient, since pronuclei detection allows for analyzing only fertilized oocytes, reducing costs and time wasting. Furthermore, combining the first and second PB biopsy could result in an improved abnormalities detection rate [25].

However, the PB biopsy only provides maternal genetic information and does not consider parental or mitotic division abnormalities [26]. European Society of Human Reproduction and Embryology started in 2012 a multicenter randomized clinical trial to establish the effectiveness of PGT-A performed with PB biopsy. The aim of the study was to evaluate whether the analysis of 23 chromosomes in the first and second polar body, and the selection of euploid embryos for transfer, increased live birth rate within one year, in women in advanced maternal age as compared to cycles without PGT-A. From June 2012 to December 2016, 205 women were assigned to cycles with PGT-A, and 191 to cycles without PGT-A (control group). However, the LB rate was not different among the two groups: 50 out of 205 (24%) in the PGT-A group and 45 out of 191 (24%) in the control group [27].

PB biopsy has the benefit of providing a long time to perform genetic testing without requiring embryo cryopreservation despite being time-consuming [25,26] and less cost-effective per LB rate [28] and, therefore, it is the only genetic testing strategy available in many countries with legal restriction on embryo genetic assessment and cryopreservation.

### 2.2. Blastomere Biopsy

Blastomere biopsy is usually performed when the embryo is made of about six or eight cells, which usually happens 72 h after insemination. The first step to perform the biopsy is to open the zona using tyrode acid, mechanical piercing, or laser-assisted hatching. Laser assisted zona drilling and the use of calcium-magnesium free media to weaken cell cohesion is the most widespread procedure according to the report of ESHRE PGT consortium in 2011 [29].

It is possible to remove one to two blastomeres. Two cells biopsy is more accurate, but it could affect embryo vitality, since it results in the removal of about 30% of the whole embryo. One cell biopsy, on the other side, could result in misleading or incorrect diagnosis [30]. Other studies [31,32] have suggested that removing of more blastomeres has negative effects on embryo development, which leads to reduced implantation rates, but it provides a higher diagnostic efficiency when compared with the removal of only one cell.

However, this technique is compatible with fresh embryo transfer on day 5 or 6 of embryo development, given that genetic results will usually be available one to two days after blastomere biopsy [31].

### 2.3. Trophoectoderm Biopsy

The blastocyst is composed of two different cell types: the inner cell mass, which will evolve in fetal tissues, and the trophoectoderm (TE) considered the precursor of future placenta. The advantages correlated to TE biopsy are mainly three: first of all, TE is not involved in fetus formation, as it will form extra-embryonic tissues. The second benefit is that blastocyst stage embryos have already activated their genome, allowing for a more accurate analysis [25]. Finally, a sample of about five-eight cells is needed for the test, determining a loss of about 10% of all of the cells forming the blastocyst (about 100–150). When compared to the cell mass loss determined by the removal of two blastomere, this procedure seems to be much less invasive [33]. Blastocyst biopsy also implicates other practical advantages: embryos vitrified at this stage show a higher survival rate if compared with cleavage stage embryos. Therefore, it allows for postponing the transfer and even adopt a single embryo transfer policy reducing multiple pregnancies [34].

**Figure 1:**
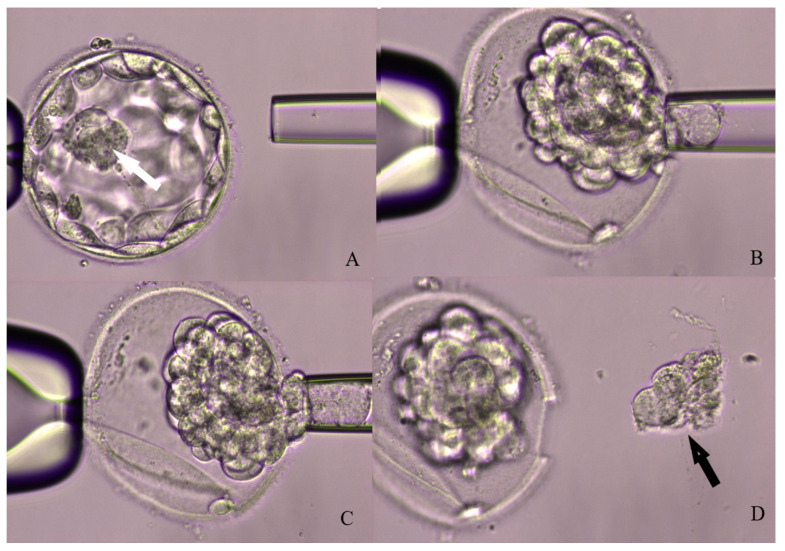
In the figure, the sequence of a blastocyst biopsy laser assisted is shown. The blastocyst is initially orientated by mean of the holding pipette in order to keep the inner cell mass as far as possible from the site of biopsy (**A**). Subsequently, the biopsy pipette is introduced through a hole performed with laser in the zona pellucida and a little number of trophectoderm cells are gently aspirated (**B**,**C**). Finally, the removed trophectoderm cells is transferred in a tube for the genetic analysis (**D**). The white narrow indicates the inner cell mass. The black narrow indicates the removed trophectoderm cells.

Three main approaches can be followed for TE biopsy: the first consist in opening the zona pellucida at cleavage stage using a laser-assisted drilling and then waiting for the formation of expanded or herniating blastocysts on day 5 [35]. Cleavage-stage zona drilling is performed to obtain a faster biopsy on herniating blastocysts and reduce the chance of sudden collapse. Although being widely adopted, this procedure presents two main limitations; it entails two sessions of embryo manipulation outside the incubator and there is the concrete risk of having the inner cell mass herniating outside the zona. The second approach to TE biopsy is to leave the embryo in culture until blastocyst full expansion and then open the zona immediately before the biopsy, with assisted laser hatching. This strategy requires a single intervention on the embryo and the zona can be opened in a region far from the inner cell mass, reducing its involvement in the biopsy process. The last method takes advantages of both the previous approaches: it consists of opening the zona when the blastocyst is fully expanded and then waiting for the TE herniation. Figure 1 shows the blastocysts biopsy laser assisted steps.

It has been demonstrated that the biopsy protocol might affect clinical outcomes [36]. The approach entailing sequential hatching and biopsy results in a significantly higher survival rate after thawing, implantation, clinical pregnancy, and LB rate if compared to the cleavage stage hatching approach. However, day 3 pre-hatching, extends the time of exposure outside the incubator and the risk of having a blastocyst herniating from the inner cell mass requiring extra manipulation during the biopsy. Furthermore this procedure allows a better synchronization with the natural expanding process of the blastocysts that could take place on day 5, 6, or 7. This technique is also cost-effective, since leaving the embryo undisturbed from fertilization to blastocyst formation allows for the employment of single-step media and time-lapse incubation protocol. 

Another controversial theme regarding TE biopsy is whether day 6 and day 7 blastocysts should be analyzed or not. A study by Piccolomini and co-workers [37] investigated if slow development might reflect embryo ploidy status. This group compared blastocyst biopsy performed on day 5 versus day 6 and reported similar aneuploidy rate (61.4% on day 5 vs. 69.9% on day 6). The study by Taylor et al. [38] evidenced that day 5 blastocysts had a significantly higher chance of being euploid than day 6 blastocysts (54.6% vs. 42.8%). Both of the studies concluded that blastocysts formed on day 6 and have the same chance of resulting in a live birth rate as those formed on day 5. The study by Hernandez-Nieto et al. [39] found that the rate of embryo euploidy was significantly lower in day 7 blastocysts when compared to day 5 or day 6 cohorts (40.5% vs. 54.7% vs. 52.9%, respectively). In his study there was also a significant decrease in the odds of implantation, clinical pregnancy, and LB, but no association with pregnancy loss in patients who transferred day 7 biopsied euploid blastocysts.

Although day 5 blastocysts may have the higher euploid rates, its relationship with embryo development is still unclear [4,40,41]. On the other hand, day 7 blastocysts can be viable, of top morphology, euploid, and result in a healthy live birth. Therefore, culturing embryos an additional day increases the number of embryos useable per IVF cycle and provides further opportunity for patients who have only a few or low-quality blastocysts. These findings underlined the importance of performing biopsy of all blastocysts available independently of their morphology or growth-timing.

### 2.4. Non-Invasive PGT

Embryo biopsy, performed at every developmental stage, is an invasive process that might condition IVF results. There are two alternatives to invasive biopsy: blastocentesis, consisting in the analysis of the blastocyst fluid (BF), and the examination of the spent culture media. The sampling of BF is performed on the opposite side of the inner cell mass, leaving the embryo fully collapsed [42,43]. Because dynamic collapse and re-expansion of the cavity is a phenomenon routinely observed during laboratory practice, the loss of the BF should not be detrimental to the embryo [44,45]. The aspiration of the BF does not affect embryo architecture, which results in high survival rates of both good and poor morphology embryos [46].

In 2013, Palini et al. [47], using real-time PCR, reported the presence of DNA fragments in BF obtained from day 5 blastocysts. The investigation of these DNA fragments allowed for the identification, with a 95% accuracy, of male embryos, detecting the specific Y-linked protein. Another study, conducted in 2015 by Tobler et al. [48], analyzed BF from 96 embryos: embryonic DNA was recovered and analyzed, using whole genome amplification (WGA), followed by aCGH in 63% of the samples. The results were concordant with those of the matched inner cell mass karyotypes only in 48.3% of the analyzed embryos. This induced the authors to recommend not to use blastocentesis as an alternative approach for PGT. Therefore, the failure of amplification rates after blastocentesis are a lot much higher if compared with those of the traditional TE biopsy [49].

On the contrary, Gianaroli et al. [50] reported the detection of embryonic DNA in 76.5% of the samples, with a diagnosis concordance rate of 97.4%, when compared to the correspondent TE biopsy. Although the analysis of BF seems to be a promising alternative to invasive PGT, further studies must be conducted. It is important to establish whether the loss of the BF could alter cell to cell communication, or the communication of the embryo with its environment. Furthermore, it is still unknown if the DNA material obtained from the blastocentesis is representative of the embryo DNA.

The analysis of spent culture media is another non-invasive alternative to traditional PGT. It consists in the analysis of cell free DNA that can be found in the media, due to its release from the embryo after apoptosis, necrosis or active release as macrovescicle. The DNA detected might have different origins: embryonal, paternal, maternal or extra-DNA. This technique does not require any experienced embryologist, since the embryo remains untouched, but it may be affected by several variables: culture drops volume, group or single embryo culture, the use of sequential or single-step media and fresh or thawed embryos culture [51,52].

A study conducted by Ho et al. [51] in 2018, compared PGT-A on spent culture media before and after blastulation, to determine the best timing for the collection of the media. The media was collected both on day 3 and day 5: DNA was detected in about 97% of samples that were collected on day 3, but only 39% of the samples provided enough material to yield a PGT-A result. On day 5, an amplification was obtained in 97% of the samples, of which 80% produced enough material for a reliable PGT-A. This study suggests the existence of an optimal collection date, to obtain more DNA quantity and a more solid analysis. The main risks that are associated with this kind of PGT-A are gentic contamination (maternal, paternal or lab personnel), the higher rate of apoptosis in aneuploid embryos, and its dependence on laboratory routine.

Many studies have been conducted in the last years to evaluate this technique and many of them reported moderate success rates [51,52,53]. Although very promising, minimally or non-invasive techniques for analyzing embryo ploidy are still at a premature stage, needing further study and standardization protocols.

## 3. PGT Molecular Techniques 

The aCGH technique allows for detecting variations in the number of copies and rearrangements of each of the 24 chromosomes when comparing the biopsied genetic material with a reference sample. After amplification by WGA the sample is labelled with fluorescent probes and hybridized to a DNA microarray. The color adopted by each spot after hybridization allows for identifying chromosomal loss or gain. A laser scanner and a data processing software are used to detect fluorescence and analyse aneuploidy and chromosomal rearrangements [54].

Single Nucleotide Polymorphism Array (SNP) is performed using an array setup consisting in DNA hybridization, fluorescence microscopy, and solid surface DNA capture. SNP found in the analyzed sample are compared with SNP of maternal and paternal derivation to assess the ploidy status [55].

Real Time Quantitative Polymerase Chain Reaction (rtq-PCR) is an assay based on polymerase chain reaction; it can identify the whole chromosome asset detecting the copy number of each chromosome. To determine the copy number, it compares three or four locus-specific amplicons along each chromosome to a reference gene from the same chromosome. It is unable to identify chromosome aberrations and uniparental disomy, but can identify triploidy [56]. 

Next Generation Sequencing (NGS) is the latest approach for pre-implantation genetic testing. The first step of this protocol consists in WGA as for aCGH. After genome amplification, a bar-coding procedure takes place, in which different samples are labelled with specific sequences. This process allows for combining from 24 to 96 biopsies (depending on the sequencing platform adopted) in a sequencing run and this optimized cost per sequenced embryo. Each sequence is then compared to a reference human genome and a specific software is used to identify copy number variations and large deletions or duplications [57,58,59].

The availability of different molecular techniques opened a debate about their sensitivity and reliability for PGT. The study by Friedenthal et al. documented that the implantation and ongoing pregnancy/live birth rate were significantly higher in women undergoing NGS (71,6% and 62,0%, respectively) as compared to those evaluated by aCGH (64.6% and 54.4%, respectively). Moreover, the comparison between NGS and aCGH group documented significantly less biochemical pregnancies (8.7% vs. 15.1%) and a similar rate of spontaneous abortion (12.4% vs. 12.7%). Based on these findings, the authors concluded that PGT using NGS significantly improves clinical outcomes with respect to the aCGH technique and suggested that NGS could be more effective in identifying mosaic embryos and those with partial aneuploidies or triploidy [60].

Friedenthal et al. [61] investigated clinical error rates in frozen-thawed embryo transfer (FTET) cycles of single euploid embryo diagnosed by NGS (1151 cases) and aCGH (846 cycles). The clinical error rates in the NGS cohort resulted in being lower with respect aCGH: 0.7% vs. 1.3% per embryo, 1% vs. 2% per pregnancy with gestational sac, 0.1% vs. 0.4% per ongoing pregnancy/live birth rate, and 13.3% vs. 23.3% per spontaneous abortion. Therefore, although NGS and aCGH are highly sensitive methods for PGT, clinicians must still consider the chance of errors occurring.

## 4. Current Insications for PGT

### 4.1. Advanced Maternal Age 

Chromosomal abnormalities in oocytes and embryos constantly increase with maternal age and reduced ovarian reserve [20,62]. However, a significant proportion of aneuploid embryos presents a good morphology [9,16,63,64,65,66] and many recent studies report that good quality blastocysts can result in aneuploid embryos in up to 44.9% of the cases [20,67,68].

A study by Franasiak et al. [20], based on more than 15,000 TE biopsies, demonstrated that the aneuploidy rate was about 20–27% between the ages of 26 and 30. Chromosomal abnormalities increased steadily from age 31 through age 43, and aneuploidy rate was then stabilized at approximately 85%. The no-euploid rate was lowest and equivalent in the embryos that were obtained from women aged 26–38 year, but a statistically significant increase of this rate was noted for successive age group. Jiang et al. [69] divided patients undergoing PGT-A into three groups according to Anti-Müllerian hormone (AMH) level: <1.50 ng/mL, 1.50–5.60 ng/mL, and ≥5.60 ng/mL. There was a significant difference in aneuploidy rate between the three AMH groups (66.7% vs. 42.9% vs. 50.0%, respectively). After age stratification, the aneuploidy rate was still significantly different among AMH groups with a similar trend in women ≥35 years old, suggesting that low AMH level was associated with increased risk of embryo aneuploidy only in women of advanced age.

In women with advanced maternal age (AMA), therefore, PGT-A assessment should be considered [69,70,71]. The most recent studies showed that embryo selection through aneuploidy screening significantly increases the chance for implantation and decreases miscarriage rate [67,72]. Being able to select the euploid embryo with higher implantation potential allows for limiting the number of embryos transferred per cycle, decreasing the chance of twins and high order multiple gestation [73]. Moreover, some studies have shown that when an euploid embryos is transferred, the implantation rate remain similar, regardless of increasing maternal age [74,75].

Rubio et al. [76] published the results of a multi center randomized two-arms trial: a PGT-A group undergoing 24-chromosome screening on day-3 embryos, followed by blastocyst transfer, and a control group without PGT-A, in which blastocyst transfer were developed in women with advanced maternal age (38–41 years). In PGT-A group, 78.6% of embryos were aneuploid, a total of 37 pregnancies were achieved with only one clinical miscarriage, resulting in a delivery rate per transfer of 52.9%. In contrast, a total of 41 pregnancies were obtained in the control group, with 16 miscarriages, and a delivery rate per transfer of 24.2%. The authors observed a significantly lower miscarriage rate in the PGT-A group (2.7% vs. 39.0%) and a reduced time to achieve pregnancy (4.5 weeks vs. 5.8 weeks) when compared to the control group.

Sacchi et al. [77], in an observational-cohort study with two years follow-up, including a total of 2538 couples, confirmed that PGT improves clinical outcomes in patients with AMA when compared to controls, as documented by higher LBR (40.3% vs. 11.0%), reduced pregnancy loss (3.6% vs. 22.6%), and multiple pregnancy rate (0% vs. 11.1%). Multivariate analysis showed no negative impact of PGT-A related interventions on the cumulative delivery rate and on neonatal outcomes.

Extensive counseling based on biological and clinical data should be provided to women older than 43 years, because of their very low odds of success and high risk for embryo aneuploidies. Ubaldi et al. [78] evaluated the efficacy of PGT-A in women older than 44 years with a good ovarian reserve. Blastocyst development was obtained in 102 (68.0%) out of 150 cycles, but only 21 (14.0%) of them resulted in being euploid. Specifically, no euploid blastocyst was found in patients older than 46, whereas the euploidy rate was 14.4% and 4.5% in the group aged 44.0–44.9 and 45.0–45.9, respectively. The delivery rate was 57.1% per transfer. However, the delivery rate per cycle was 10.6% in patients aged 44.0–44.9 years and 2.6% in patients aged 45.0–45.9 years. 

Lee et al. [79] evaluated the efficacy of PGT in women with an age included between 40 and 43; they compared the pregnancy outcomes from traditional fresh IVF cycles with day 5 embryo transfers, FTET cycles without PGT, and PGT-FTET cycles (only euploid embryos). The implantation and LB rate in PGT-FTET cycles (50.9% and 45.5%) was significantly higher than for unscreened embryos transferred in fresh (23.8% and 15.8%) or no PGT-FTET (25.4% and 19%) cycles. There were significant differences in live birth rate per embryo transferred for the three groups: 45.5% for PGT-FTET, 15.8% for fresh transfers, and 19% for No-PGT FTET. The incidence of pregnancy loss was 38.1% for fresh cycles and only 10.7% for PGT-FTET.

Outcome data coming from a total of 8175 SETs after PGT-A and embryo cryopreservation [80] evidenced that age-related decline in reproductive efficiency can be reduced selecting euploid embryos suitable for the transfer. However, the implantation rates are negatively correlated with maternal age and, also after adjusting for confounders, women 38 years or older had a significantly lower implantation rate than those under 35. These differences were also highlighted in clinical pregnancy and LB rates. Therefore, the observed impact of aging is not correlated with embryos ploidy.

Taking into considerations all of these factors, it can be affirmed that, in the AMA patient, the influence of embryo aneuploidies on infertility is relevant, and both the safety of the procedure and the reduction of the time to pregnancy achievement are of foremost importance. Patients undergoing PGT-A must be counseled that the proportion of embryos likely to be aneuploid grows in age-specific ranges, evidencing the possibility of obtaining only aneuploid embryos. It should also be noted that obtaining one euploid blastocyst becomes more challenging with increasing maternal age, since there are many adverse factors that are associated with cycle cancellation, such as the lack of follicular development, unsuccessful oocyte retrieval, and lower blastocyst formation rate.

### 4.2. Recurrent Pregnancy Loss 

Approximately 5% of all couples undergoing IVF treatment are affected by recurrent pregnancy loss (RPL), defined as two or more consecutive miscarriages at a gestational age up to 20 weeks. It can be explained by genetic, anatomic, endocrinologic, and immunologic abnormalities [81]. However, in more than 50% of cases, the current diagnostic procedures are not able to identify etiologic factors [82,83,84]. Unexplained RPL is a distressing condition for the affected couple, and a frustrating problem for the clinician since no effective therapy exists. 

A retrospective cohort study including 46,939 women who underwent fetal karyotype analysis through amniocentesis or chorionic villus sampling, documented that women with no prior spontaneous abortions had a risk for aneuploidy of 1.3%. In women with one, two, or three previous spontaneous abortion this risk increased significantly to 1.6%, 1.8% and 2.1%, respectively [85]. A higher aneuploidy rate in RPL patients has also been confirmed by other authors [83,84,85,86,87].

PGT-A could be helpful in the treatment of couples with unexplained RPL, when considering that embryo aneuploidies could be the cause of miscarriages. Several studies using genetic testing in couples with this indication have shown a decrease in miscarriage rate [88]. Earlier studies were typically performed with the use of FISH on cleavage-stage embryos and typically tested only 7–12 chromosomes. In one meta-analysis [89], four observational studies [70,90,91] evaluated patients with RPL undergoing cleavage-stage biopsy and compared them to patients looking for a natural-conception. The spontaneous abortion rate of 9% in the first group was significantly lower than in the controls (28%).

The incidence of aneuploidies in blastocysts from patients with idiopathic RPL undergoing PGT-A and PGT for monogenic diseases (PGT-M) resulted to be increased in women aged ≤35 years (48.9% vs. 36.9%), whereas no significant increase was found in group aged >35 years (66.9% vs. 61.4%). However, despite euploid embryo transfer, young patients had a higher miscarriage rate (26.1% vs. 3.1%) suggesting that in these group of patients RPL may derived not only from genetic reasons [92]. 

A literature review searching for available evidences on LB and miscarriage rates after PGT-A, compared to natural conception in couples with unexplained RPL suggested that PGT-A application, might reduce the miscarriage rate when compared to natural conception (9% vs. 28%) [89]. Hodez-Wertz et al. [93] documented that, in a total of 2282 embryos analyzed, 60% were aneuploid. Euploid embryo transfers performed were 181, with an implantation rate of 45% and ongoing pregnancy rate of 92%. The miscarriage rate was found to be only 6.9%, as compared with the expected rate of 33.5% in a RPL control and 23.7% in infertile control population. Therefore, the authors concluded that PGT-A by aCGH decreases miscarriage rate in idiopathic RPL, providing, at the same time, diagnosis and treatment for these patients.

No significant differences in LB and miscarriage rates per patient given or not given PGT-A were evidenced by Sato et al. [94]: 26.8 versus 21.1% and 14.3% versus 20.0%, respectively. Live birth rate per embryo transfer was improved by the application of PGT-A (52.4 vs. 21.6%), which also had the advantage of reducing the number of embryo transfers that are required to achieve a similar number of live births as compared with cycles in which PGT was not performed.

In the study by Murugappan et al. [95], PGT-A in patients with RPL resulted in a LB rate of 53% and a clinical miscarriage rate of 7%. Expectant management had a LB rate of 67% and clinical miscarriage rate of 24%. However, the IVF/PGT-A strategy was 100-fold more expensive, costing $45,300 per live birth when compared with $418 per live birth with expectant management. According to current literature, IVF/PGT-A is a very expensive way to reduce miscarriage, without increasing the chance of achieving a live birth; further study should be conducted before recommending it as standard treatment for RPL.

Katz-Jaffe et al. [96] showed a higher incidence of aneuploid blastocysts in patients with diminished ovarian reserve (DOR), whereas Trout et al. [97] evidenced a higher incidence of DOR in RPL patients. Shahine et al. [98] examined the role PGT-A in patients with RPL and DOR, being defined as a cycle day 3 FSH > 10 IU/mL and/or antimüllerian hormone <1 ng/mL. Patients with DOR had a higher percentage of aneuploid blastocysts (57% vs. 49%) when compared to controls. In all patients aged <38 years, a higher aneuploidy rate of was observed in embryos from DOR patients, when compared to patients with normal ovarian reserve (67% vs. 53%, *p* = 0.04). In the two groups, the implantation and the live birth rates after transfer of euploid blastocysts were similar (61% vs. 59% and 54% vs. 47%, respectively), despite the difference in ovarian reserve testing. The miscarriage rate was slightly higher in the DOR group, but it was not statistically significant (14% vs. 10%). It can be affirmed that patients with RPL and DOR, transferring euploid embryos, may benefit from PGT-A, as demonstrated by outcomes that are comparable with those of patients with RPL and normal ovarian reserve testing.

### 4.3. Repeated Implantation Failure 

Repeated implantation failure (RIF) is defined by ESHRE PGD Consortium, as the absence of a gestational sac on ultrasound at five or more weeks after three embryo transfers with high quality embryos or after the transfer of ≥10 embryos in multiple transfers [99]. RIF is not only distressing for patients who require multiple cycles of treatment, but it also increase the costs of IVF procedure. This condition is still a big challenge to the clinician, since it could have multiple causes, depending on maternal or embryonic factors [100,101,102]. Although not all RIF cases are due to embryonic defects, many patients have different chromosomal abnormalities [103] and the extension on these anomalies, increases with the number of previous failed IVF cycles [102]. A high level of chromosome abnormalities is present in arrested embryos, but also in embryos with good morphology [11,15,20,67]. Several approaches have been used in RIF patients to select a good embryo for the transfer, such as blastocyst culture [104] or assisted hatching, which seems to improve embryo implantation capability [105].

Many centers started to perform PGT-A on single cells biopsied from cleavage stage embryos using FISH with chromosome-specific probes [106,107]. A study by Rubio et al. [71] aimed to evaluate the role of PGT by FISH for two different indications: women <40 years with RIF and women with AMA aged 41–44 years. The patients were allocated in two arms: blastocyst transfer without PGT or PGT followed by day 5 embryo transfer. In the AMA group, a significant increase in live-birth rate per patient was found in PGT group when compared with no-PGT group (32.3% vs. 15.5%). However, in RIF patients, a trend toward higher LB rate was noted (47.9% vs. 27.9%), but without statistically significant difference. 

Comparative genomic hybridization is a molecular cytogenetic technique that can be applied to single cells in inter-phase to allow simultaneous analysis of every chromosome, in contrast with FISH. However, the study by Voullaire et al. [108] including women aged 26–41 years with RIF showed that 40% of the embryos could be considered suitable for transfer, a value that is similar to that found using FISH for PGT-A (ESHRE PGD Consortium Steering Committee, 2002) [109]. Infertility in this group of patients is likely to be multi-factorial and chromosomal abnormalities could not be involved in it [108]. 

The aim of the study performed by Greco et al. [110] was to assess the clinical pregnancy and implantation rates, after transferring a single euploid blastocyst tested with aCGH, in a group of patients younger than 36 years with a history of RIF. These results were compared with a similar group of RIF patients in whom PGT was not performed and with a group of good prognosis patients after PGT. The euploidy rate in RIF PGT and in NO RIF PGT were 46.2% and 51.8%, respectively. Clinical pregnancy and implantation rates, respectively, were 68.3% and 68.3% in RIF PGT, 22.0% and 21.2% in RIF NO PGT, and 70.5% and 70.5% NO RIF PGT. There were no spontaneous abortions in any group. The results from RIF NO PGT were significantly lower when compared to other two groups.

The similar clinical results after single euploid blastocysts transfer in good prognosis and RIF patients at their first IVF attempt demonstrated that embryo aneuploidies may be the most relevant cause of implantation failure [110].

### 4.4. Male Factor Infertility 

Although embryonic aneuploidies mainly arise from maternal genome, some aneuploidies may derive from the spermatozoa [21]. Males with abnormal karyotype and Y chromosome deletions tend to produce spermatozoa with an unbalanced chromosome complement. Other several factors, such as varicocele, chemotherapy, age, and lifestyle, may also negatively affect meiotic divisions during spermatogenesis [21]. The rate of abnormal sperm after FISH examination was significantly higher in patients with male infertility (55.8% vs. 15.0%) and teratozoospermia was highly correlated with aneuploidy rate for chromosome 17, as documented by Petousis et al. [111]. Therefore, PGT-A should be considered in ICSI cycles with severe male factor (SMF), including azoospermia (obstructive and non-obstructive), severe oligoastenoteratozoospermia, Klinefelter syndrome (KS), Y-chromosome microdeletion, and even in men whose semen analysis does not fulfill the current World Health Organization (WHO) criteria.

Magli et al. [112] showed that SMF might contribute to a higher rate of aneuploid blastocysts (55% aneuploidy rate with normozoospermia, 62% with oligozoospermia, and 69% with nonobstructive azoospermia (NOA)), as evidenced by the 9-chromosome FISH technique performed on day-3 embryos. In a study by Silber et al. [113], patients with oligozoospermia and azoospermia had an euploidy rate of 41% and 22%, respectively, but the analysis was still conducted with the use of 9-chromosome FISH. Coates et al. [114] performed aCGH on TE biopsies; a significant increase in sexual chromosome abnormalities was observed in oligozoospermic males both with own (6.1% vs. 5.9%) and donor (1.7% vs. 2.0%) eggs.

A cohort study based on 1219 consecutive ICSI cycles performing PGT with trophoectoderm biopsy using rtq-PCR [115] evidenced that the rate of euploid blastocyst per inseminated mature oocyte was only significantly reduced in NOA (11.1%) when compared with normozoospermic patients (16.3%), whereas the euploidy rate per biopsied blastocyst was similar among the two groups. Furthermore, no differences in gestational age and birth weight were reported among the groups. The overall prevalence of congenital malformations (2.1%) was similar to that previously reported after either IVF with and without PGT-A [116,117] or spontaneous conceptions [118,119]. Following all of these considerations, SMF was not considered to be a critical parameter for embryo ploidy status. This could be due to the intrinsic potential of the oocyte to prevent further development of aneuploid embryos before the activation of the embryonic genome [120] or to the fact that sperm-derived aneuploidies may result in an early interruption of embryo development [121].

Mazzilli et al. [115] investigated in their study only 35 cycles in women under 35 years. Kahraman et al. [107] investigated 326 cycles with 741 blastocysts biopsied and analyzed using aCGH and NGS techniques; the couples enrolled were composed of young female (≤35) with SMF partners, ranging from five million/mL to NOA.

The couples undergoing IVF cycles with testicular spermatozoa had higher aneuploidy rate with respect to the patients with SMF and controls. In addition, reduced spermatozoa count (1–5 mil/mL) was associated with aneuploidy rate of 15.6%, suggesting that SMF increases the risk of chromosomal abnormalities, regardless of the female age. The highest mosaicism rate (22.0%) was observed in cycles using testicular spermatozoa and it was significantly higher than in cycles with normal sperm parameters (9.9%). No significant difference was observed in the clinical outcomes between the control group and the SMF groups after transfer of a single euploid embryo. There was a trend toward a higher miscarriage rate in testicular sperm group.

A retrospective analysis by Tarozzi et al. [122], in which blastocysts from 340 cycles were assessed by aCGH, no differences between male (MF) and non-male factor (NMF) groups were found in terms of euploid blastocysts rate. The MF group showed a significantly higher rate of mosaic embryos (3.6% vs. 0.5%, respectively). A similar pattern of results was observed in all SMF groups considered in their complex, when compared to those without SMF (7.7% vs. 1.8%), suggesting that a compromised semen quality is associated with an increase in the mosaic blastocysts rate. The reason of these differences could be found in the fact that, during spermiogenesis, the transit of the spermatozoa in the epididymal tract favors DNA packaging, through the stabilization of the chromatine structure with protamine dephosphorylation and the formation of intra and intermolecular disulphide bridges between protamines.

The study by Scarselli et al. [123] evaluated whether the duration of ejaculatory abstinence might influence the euploid blastocysts rate. The blastocysts euploidy rate resulted in being 27.5% using spermatozoa from semen sample obtained after 2–5 days of abstinence and of 43.5% using spermatozoa from the second sample obtained 1 h after the first one. The use of potentially higher quality spermatozoa from samples with a shorter abstinence might reduce the aneuploidy rate in blastocysts.

Non-mosaic Klinefelter syndrome is associated with a male genetic disorder that often leads to NOA. The literature reports about 101 children born from non-mosaic KS fathers after successful ICSI, in most cases, healthy and without the use of PGT to select normal embryos [124]. Fetuses or embryos were diagnosed to have 47,XXY karyotype in some cases [125,126]. Due to these contradictory findings, it remains an open question as to whether embryo biopsy should be offered to couples with KS male partner. In the study by Greco et al. [127], a total of 26 ICSI cycles were performed in couples with KS and a total of 11 pregnancies and deliveries were achieved (pregnancy rate 42.3%). All of the fetal karyotypes were normal. PGT-A was not done, and the embryo chromosomal assessment was unknown at the time of transfer. It could be thought that, when the SMF is due to altered karyotype, it does not affect embryo ploidy. However, Staessen et al. [128] published an extremely convincing study on the need for PGT in KS patients. They reported data on PGT-M offered to 20 non-mosaic KS, showing that 54% of the embryos were normal but KS patients had a higher incidence of abnormalities involving chromosomes 18 and 21.

Several studies have also explored the effect of advanced paternal age on sperm aneuploidy rates. Two recent studies [129,130] noted that males above the age of 50 present more sperms with damaged DNA, low blastocyst development rate, higher global aneuploidy rate, and a higher rate of trisomies. Advanced paternal age could lead to several alterations in endocrinal and reproductive phenotypes, leading to an increase in DNA damages over years and a decrease in germ cells capability to repair these damages, inducing the production of aneuploid sperms and, consequently, abnormal embryos [131].

The study by Gat et al. [132] evaluated 177 IVF-ICSI cycles, in which PGT-A was performed on 405 blastocysts and concluded that DNA fragmentation index (DFI) was not associated with impaired embryo quality in both genetic and morphological aspects. Similar DFI-related findings were published by Bronet et al. [133], who reported comparable embryo euploidy rates in different DFI levels.

Overall, approximately 25–55% of males with extreme testicular pathologies, such as hypospermatogenesis, sperm maturation arrest, and Sertoli cell only syndrome (SCOS), and about 5–25% males with severe oligozoospermia or azoospermia present Y chromosome microdeletions. In general, deletions of the entire AZFa region invariably result in SCOS and azoospermia. Patients with deletions of the AZFb region presents maturation arrest, mostly at spermatocyte stage. Men with AZFc deletions have the most variable phenotype, which ranges from complete azoospermia to mild oligozoospermia [134]. Comparisons between the different types of microdeletions and treatments revealed that, except AZFc, cases with AZFa and AZFb microdeletions presented a poor clinical prognosis. On 125 patients, 25 of them (20.0%) had abnormal karyotypes. Ninety-four cycles with testicular sperm leaded to 19 deliveries. Twenty-nine cycles with ejaculated sperm leaded to eight births (all in couples with AZFc). Patients with such alterations should be informed about the possibility of using preimplantation genetic testing due to the reported heredity of microdeletions and a possible association with men abnormal karyotype [134].

### 4.5. PGT in a Good-Prognosis Patients Undergoing SET 

Choosing the best embryo to transfer is crucial, especially when a single embryo transfer program is adopted for different clinical reasons [135]. The first study to prospect a successful elective SET after a rapid on-site aCGH application was performed by Yang et al. [67] in good prognosis women <35 years of age. Fifty-six patients were randomized in two groups: in the first one a morphological evaluation of the embryos was used to select the one for the transfer in combination with aCGH, in the second one, morphology was used as the only discerning parameter. The aneuploidy rate in 425 blastocysts analyzed with aCGH was 44.9%, whereas 389 blastocysts were microscopically examined in the control group. The clinical and ongoing pregnancy rates were significantly higher in the morphology plus aCGH group as compared to controls (70.9 vs. 45.8%, and 69.1 vs. 41.7%, respectively). No twin pregnancies occurred in both groups. A low miscarriage rate was noted for all of the study patients, although this was slightly lower in the PGT-A group (2.6 vs. 9.1%).

Despite an increasing acceptance of elective SET treatment, many IVF cycles continue to involve the transfer of two or more embryos. Scott et al. [32] evaluated whether blastocyst biopsy with rtq-PCR comprehensive chromosomal screening might improve IVF outcome in women under 42 years with normal ovarian reserve. The aneuploidy rate was 28% among patients who were included in the genetic testing group. Clinical implantation rate and the proportion of screened embryos that progressed to delivery (79.8% and 66.4%, respectively) were significantly higher when compared to the control group (63.2% and 47.9%, respectively).

A recently published randomized clinical trial by Ozgur et al. [136] divided 220 patients aged ≤35 years in an arm, in which a single euploid blastocyst was transferred and an arm in which single unknown-ploidy blastocysts were transferred. In the PGT-A group, 73.4% of all blastocysts were diagnosed as euploid, suggesting that the best-scoring blastocysts of the selected infertile patient (≤35 years) group had the same probability to be healthy. The live birth rate in euploid subgroup was found not to be statistically different as compared to morphology group (56.3% vs. 58.6%), which suggests that PGT-A is not able to enhance LB rate in young patients [136].

Munne et al. [137] draw the same conclusions in a study in which a total of 661 women (average age 33.7 ± 3.6 years) were randomized to PGT-A or morphology only group. The ongoing pregnancy rate was equivalent between the two arms, with no significant difference per embryo transfer (50% vs. 46%). Post hoc analysis of women aged 35–40 years showed a significant increase in the ongoing pregnancy rate per embryo transfer (51% vs. 37%), but not per intention to treat. 

In a study conducted by Forman et al. [138], 43.4% of the 175 randomized patients were <35 years old, 30.9% were 35–37 years old, 19.4% were 38–40 years old, and 6.3% were 41–42 years old. The different groups showed the same clinical performance. The ongoing pregnancy rate after each patient’s first transfer, whether fresh or frozen, was 60.7% after single euploid blastocyst transfer and 65.1% after two untested blastocyst transfer. It must be underlined that patients who received single euploid blastocyst transfer were nearly twice as likely to have an ongoing singleton pregnancy when compared with those with two blastocysts transfer (60.7% vs. 33.7%). This trial demonstrates that the singleton delivery rate can be improved while using a validated method to assess embryo ploidy, without compromising the overall success rate.

The transfer of single euploid blastocyst might prevent twins and higher order multiple pregnancies reducing the costs and increasing the efficacy and safety of IVF procedure. However, a higher risk of monozygotic twinning following assisted reproduction procedures (1.4%) as compared with natural conception (0.4%) has been evidenced [139]. Some studies showed a consistent association between the extended embryo culture and embryo splitting [140]. However, it is still unclear as to whether embryo biopsy represents a risk factor for monozygotic twinning [141,142].

In a retrospective cohort study from a large referral center in Belgium, the incidence of monozygotic twin births following PGT was 1.5%, and 2.1% following ICSI cycles without PGT [143]. In addition, also a systematic review, pooling results from four studies, reported no increased risk of monozygotic twinning after PGT as compared with IVF without PGT [144]. However, Kamath et al. [145] evaluated data from 207,697 SET cycles being performed mainly in women aged <35 years with no infertility diagnosis; many of the cycles were performed as part of a PGT-M or PGT-SR program. There was a significantly higher risk of zygotic splitting with PGT versus non PGT IVF cycles (2.4% vs. 1.5%), even after adjustment for potential confounders. Such contradictory findings could be due to different embryo biopsy techniques adopted in these studies (for example, cleavage stage biopsy versus trophoectoderm biopsy).

### 4.6. PGT-A in Donor Egg Cycles 

Donor eggs are usually collected from healthy, young, and fertile women, hoping that most eggs will have normal chromosomal integrity. When considering literature data presented in the previous chapters of this review, it can be affirmed that even young women produce a significant proportion of aneuploid embryos and screening out such abnormalities could potentially increase the efficacy of donor egg (DE) cycles [146].

According to the Society of Assisted Reproductive Technology, live birth rate in patients who undergo egg donation programs is only 5–10% higher than in patients under 34 years who use their own eggs. Embryos aneuploidy is an important factor affecting clinical success in young patients undergoing IVF [67] and, therefore, it might also affect embryo implantation in ovodonation cycles. In fact, it has been reported that the aneuploidy rate of embryos obtained from donor eggs might reach 53.2% and 88.1% of the embryo aneuploidies are of a maternal source, suggesting the necessity of PGT-A in DE cycles [147].

On the other hand, in the study by Hoyos et al. [148], oocyte donors aged ≤25 had similar blastocyst formation and ploidy rates. No correlation was found between euploid blastocyst rate and donor age. There is no need to favor a specific age subgroup of young oocyte donors, given the lack of significant age-related change in blastocyst euploid rates.

Haddad et al. [149] observed that, in DE cycles with PGT-A, 39.1% of blastocysts were abnormal. The transfer of normal euploid blastocysts lead to a clinical pregnancy rate of 72.4%, an ongoing/delivery rate of 65.5%, and an implantation rate of 54.9%; these rates were slightly higher than those in the control group (66.7, 54.0, and 47.8%, respectively), but no significant difference was found. Miscarriage rate was higher in the control group (19.2%) than in the PGT group (9.5%), but, once again, the difference did not reach statistical significance. These findings suggest that PGT-A might only be necessary in some specific situations, such as the need of a single embryo transfer. In 2019, Masbou et al. [146] compared, in a retrospective cohort study, clinical outcomes in patients undergoing ED cycle with SET with and without pre-implantation genetic testing. This study revealed that PGT-A and subsequent euploid SET did not improve pregnancy outcome in DE cycles, although there was a trend toward decreasing of miscarriage rate. Overall, these results suggest that the benefits of performing PGT-A on embryos derived from young DE may be limited. 

It is important to evaluate the potential effect of oocyte cryopreservation on embryo aneuploidies, especially for centers using vitrified oocytes in egg donation programs. Forman et al. [150] did not find significant difference in the rate of embryonic aneuploidy between fresh and vitrified donor oocytes. In this study, thirty-three patients who thawed 475 oocytes that had been cryopreserved for a median of 3.5 years, were compared with 66 age-matched controls who underwent IVF with PGT-A based on fresh oocytes. No differences were found in the percentage of euploid blastocysts (44.5% vs. 47.6%), implantation (65% vs. 65%), and live birth rate (62.5% vs. 55%) after 24-chromosome PGS with cryopreserved or fresh oocytes.

### 4.7. PGT for Monogenic Diseases

Pre-implantation genetic testing for monogenic diseases (PGT-M) is an advisable approach for couples with the risk of transmitting genetic diseases to their offspring. However, chromosomal aneuploidies can involve chromosomes that different from those that were investigated with PGT-M. The first successful attempts to perform a double factor analysis (PGT-A and PGT-M) were reported by Obradors and collaborators [151]; the aim was to improve the implantation rate selecting potentially euploid embryos free of mutations responsible for cystic fibrosis [151] or Von Hippel–Lindau syndrome [152]. However, in both case reports, first genetic screening was performed by aCGH on oocyte polar bodies for PGT-A and the second using PCR on day 3 blastomeres for PGT-M. A similar procedure was applied by Rechitsky et al. [153] in 96 cycles resulting in the transfer of 153 unaffected aneuploidy-free embryos and 32 healthy live births.

To our knowledge, the largest reported series of double genetic tests for different monogenic diseases or translocations and aneuploidy screening, performed with a single biopsy on 1122 blastocysts, was presented by Minasi et al. [12]. All of the biopsies were performed at blastocyst stage and analyzed by WGA, followed by PCR for monogenic diseases and aCGH for all cycles. The study demonstrated that, while 55.7% of the biopsied blastocysts were healthy after PGT-M analysis, only 27.5% of them were also euploid. Clinical pregnancy, implantation, and miscarriage rates that were obtained after the application of both techniques were 49.0, 47.7, and 9.9%, respectively. Without performing PGT-A in association with PGT-M, 316 blastocysts resulted in being normal or carrier for the genetic disease could have been transferred, leading to implantation failures, miscarriages, or, even, to unhealthy live births.

The value of this double screening was also explored by Goldman et al. [154] in a retrospective cohort study, including patients who underwent PGT-M with or without 24-chromosome aneuploidy screening. There were no differences between the PGT-M and aneuploidy screened group and PGT-M only group, when comparing the percentage of blastocysts affected by the single gene disorder of interest (37.0% vs. 32.8%). Despite a young mean aged population, only 25.6% of the blastocysts that resulted in negative or carriers of the single gene disorder were also euploid. In the PGT-M only group, 54.7% of embryos resulted in being suitable for transfer according to their unaffected or carrier status (*p* = 0.001). In patients undergoing both genetic screenings, the implantation rate was higher (75% vs. 53.3%) and miscarriage rate lower (20% vs. 40%) as compared to controls. The Authors concluded that PGT-M performed concurrently with 24-chromosome aneuploidy screening provides valuable information for embryo selection, and significantly improves SET rate.

## 5. Mosaicism

Mosaicism is defined as the presence of different cell lines in the same embryo. Two different kinds of mosaicism can occur: diploid/aneuploid mosaic with a mix of aneuploid and euploid cell lines and aneuploid mosaic with a mix of cell lines with different chromosomal abnormalities. There can be various types of aneuploidies in mosaic embryos: single chromosome loss or gain, complex or structural aneuploidies [155]. The origin of mosaicism is related to mitotic errors happening after fertilization at the third division stage. These mitotic errors, taking place before DNA duplication, are basically: anaphase delay, mitotic non-disjunction, accidental chromosome demolition, or premature cell division. The aneuploid cells rate depends on the time at which mitotic error happens; in embryos in which errors take place at the second cleavage stage, there will be a higher percentage of aneuploid cells [156,157]. Occasionally, mosaicism may derive from a meiotic non-disjunction event, causing a trisomic conceptus, followed by a post-zygotic event (trisomy rescue) [158,159].

FISH was first technique used to investigate the frequency of embryonic mosaicism: studies involving this method reported mosaicism rates that varied from 30% to 90% at cleavage stage, and from 18% to 46% at blastocyst stage [160]. More clear information regarding the mosaicism rate was obtained with the introduction of recent molecular techniques for genetic testing, such as SNP array, aCGH, and NGS. The mosaicism rates detected using these analyses are lower than those reported using FISH. At cleavage stage, the incidence of mosaic diploid-aneuploid varies from 15% to 71%; at blastocyst stage, the rates are lower, with an incidence ranging from 3.9% to about 33% [65,161]. Moreover, mosaic diploid/aneuploid embryos rate, decreases with maternal age: in women, less than 35 years old, the rate is 26.6%, and in >42 years old women it is 10.5% [162].

Studies regarding the distribution of mosaic cells in the blastocyst have been conducted to establish whether there is a preferential distribution between TE and inner cell mass and a recent one demonstrated that, in low-level mosaicism, there is a preferential allocation in TE, but, in high-level mosaicism, the aneuploidies seems to be uniformly distributed in the whole embryo [163]. An important question, still open, regarding mosaic embryos, is whether data obtained from a small portion of TE cells may predict the whole embryo asset. It has been hypothesized that the mosaicism rate deduced from a single TE biopsy might not be representative of the remaining TE cells or of the inner cell mass [164].

Mosaic embryos have not been considered to be suitable for transfer and they were discarded, while considering them as aneuploid embryos. Mosaicism was supposed to be responsible for altered embryo development, thus leading to implantation failure, or resulting in congenital malformation, mental retardation, and uniparental disomy [164].

Greco et al. [165] were the first, in 2015, to demonstrate that mosaic diploid/aneuploid embryos were suitable for implantation, giving birth to healthy babies. In his study, 18 diploid/aneuploid mosaic embryos, diagnosticated by aCGH, were transferred in women who did not have any euploid blastocyst for frozen embryo transfer. The implantation rate of these embryos was 44%, with a live birth rate of 33%. Every fetus was confirmed through chorionic villi to have a normal karyotype. Since then, a great number of studies involving the transfer of mosaic embryos were developed and they all confirmed the capability of these embryos to lead to healthy live births [166,167]. Table 1 reassume the clinical outcomes after mosaic blastocysts transfer presented in the most relevant papers [155,157,165,166,167].

Several studies have demonstrated that chromosomal mosaicism extent significantly affect clinical results. Spinella et al. [155] proved that blastocysts with a mosaicism rate lower than 50% had significantly higher ongoing pregnancies (40.9% vs. 15.2%) and live births (38% vs. 19%) when compared to those with a mosaicism rate over 50%. These findings were confirmed by the paper of Viotti et al. from 2019 [168]. This study assessed that complex and single segmental mosaics had better outcomes than those with multiple gain or losses of whole chromosomes [168]. Victor et al. [167] assessed that the mosaicism rate is not correlated with clinical outcomes, reporting any significant difference in embryos with mosaicism rate lower than 40% or included between 40% and 80%. Clinical outcomes were only affected by maternal age and the type of mosaicism involved, with better results in young women and segmental aneuploidies.

A still unsolved question is whether the transfer of mosaic embryos might result in congenital abnormalities or in the birth of affected babies. Over 200 pregnancies have been reported, obtained after the transfer of mosaic embryos; 42 gave birth to healthy children [155,165,167] and the other were, at the moment of the studies publication, still ongoing and apparently normal [166,167,168]. However, this number is still not high enough to draw any conclusion and future studies are needed. Due to the lack of these data, a transfer involving mosaic embryos should only be performed when unavoidable and after appropriate counseling [162]. Couples considering to transfer mosaic embryos should be informed about potential pregnancy risks and outcomes; it should be explained them that these transfers may be characterized by lower implantation and pregnancy rates, beyond a higher risk of genetic abnormalities and adverse pregnancy outcome.

It must be underlined a recently published case report: a blastocyst showing a 35% mosaicsim of monosomy 2 was transferred in a 39-year old woman with diminished ovarian reserve resulting in a clinical pregnancy. Amniocentesis revealed a mosaic trisomic karyotype: 46,XX(98)/47,XX + 2(2). This case demonstrates the need for a strict prenatal monitoring and diagnosis by early amniocentesis [169].

Preimplantation Genetic Diagnosis International Society (PGDIS) and Controversies in Preconception and Perinatal Genetic Diagnosis (CoGEN) have been suggested to always prefer the transfer of embryos with low level of mosaicism (20–40%), avoiding the transfer of those with potentially viable aneuplodies or intrauterine growth restriction [170,171].

Mosaicism rate is less than 1–2% in viable pregnancies suggesting the existence of a self-correction process that removes aneuploid cells from embryos after implantation [172]. Some models have been proposed to explain this phenomenon: one of them suggests cell death; the other a reduced proliferation of aneuploid cells when compared to euploid ones [173]. In 2019, a study by Popovic et al. [174], confirmed that, in human embryos, cell proliferation and death have different dynamics among euploid, aneuploid, or mosaic blastocysts. He used an extended in vitro embryo culture protocol to study the effect of mosaicsm on early preimplantation, up to 12 days post-fertilization. Blastocysts with high mosacism levels were more likely to be non-viable at this stage of development. This could confirm the self-correction model, since aneuploid cells could proliferate more slowly or undergo apoptosis, whilst euploid ones could proliferate faster to compensate. There is still no evidence, anyway, that could support this model.

## 6. Strategies for Euploid/Mosaic Blastocysts Transfer 

Implantation is considered to be an essential step for the success of assisted reproduction techniques and mainly depends on endometrial receptivity, embryo quality, and synchrony between them. However, the process of ovarian stimulation with elevated estrogen level, together with a possible progesterone premature growth, might reduce the expression of genes involved in the implantation process and negatively modify embryo-endometrium communication [175].

These negative effects can be responsible of decreased clinical results and adverse obstetrics and perinatal outcomes [175,176]. It has been suggested, indeed, that, after a fresh embryo transfer in a stimulated IVF cycle with E_2_ levels >2724 pg/mL at the time of hCG administration, the risk of abnormal placentation and low birth weight [177] as well as the risk of obstetric hemorrhage [178] is definitely higher.

Euploid embryos can be transferred in a fresh stimulated cycle, performing biopsy of expanded blastocysts on day 5 and waiting for PGT result for a fresh transfer on day 6. Unfortunately, slower growing embryos, which cannot be biopsied on day 5, will not be included in PGT and considered for the fresh transfer. On the other hand, the freeze-all strategy involves cryopreservation of all biopsied embryos, including blastocysts developed on day 5, 6, or also 7, and then waiting for the PGT results in preparation for a further FTET. Undoubtedly, the uncertainty of having euploid embryos available for fresh transfer might enhance stress perception, strongly present in all patients with infertility problems. At the same time, the awareness that a fresh transfer is not planned, and that the whole cohort of embryos will be carefully evaluated, might relieve patient concerns [179].

Embryo cryopreservation is often only considered as a strategy to enhance the overall pregnancy rate per oocyte retrieval, when surplus embryos are available. However, a randomized controlled trial by Coates et al. [179] compared the clinical outcomes from frozen and fresh embryo transfers, finding that where the implantation rate was similar (75% vs. 67%), the ongoing PR (80% vs. 61%) and LB rates (77% vs. 59%) were significantly higher in the frozen group with respect to the fresh one. Based on these observations, it is possible to suggest the freeze-all strategy as a routine IVF approach when PGT is performed.

Frozen-thawed embryos can be transferred in different protocols: natural cycle (NC), modified natural cycle (modified-NC), and hormone replacement cycle (HR), but the best strategy should be still identified [180]. The authors of a retrospective cohort study including 389 cycles with 24-chromosome day 5/6 PGT-A found that the ongoing pregnancy rate obtained from a single euploid FTET in NC was significantly higher when compared to that in HR regimen. No difference was documented in the miscarriage rates [181].

Several studies investigated whether the duration of estrogen therapy or progesterone values before euploid embryo transfer might influence clinical outcomes in HR programs. A retrospective cohort study suggested that the duration of estradiol treatment before progesterone initiation does not affect the immediate FTET results. However, every additional day of estrogen administration is associated with a reduction of gestational age at delivery, not exceeding the criteria of preterm birth [182].

Gaggiotti-Marre et al. [183] examined the role of progesterone levels on the day before euploid embryo transfer, still in HR protocol, and documented that patients with its values over 13.1 ng/mL have a significantly higher miscarriage rate and lower live birth rate. On the other hand, a minimum threshold of progesterone concentration must be reached on the day of ET. It was documented that the progesterone level on the day of ET was predictive of clinical success and resulted in being significantly higher (28.0 ng/mL) in pregnant women, when compared to those with negative pregnancy test (16.4 ng/mL). The optimal cut-off value suggested was at least 20.6 ng/mL [184].

At the same time, a prospective controlled randomized trial documented that HR and modified-NC protocol for FTET lead to comparable clinical results, as suggested by overall pregnancy (61.9% vs. 62.3%), clinical pregnancy (50.4% vs. 54.1%), and implantation rates (50.4% vs. 54.1%). The live-birth rate of 45.8% in modified-NC was comparable to that observed in HR (41.5%). These overlapping results suggest that the choice of endometrial preparation should be based on women menstrual characteristics or, otherwise, the need for FTET planning [180].

Nevertheless, a study conducted by Litwicka et al. observed that, in modified-NC, the timing of HCG administration for ovulation induction might be relevant. The HCG injection when LH reaches values higher than 13 mIU/mL is associated with significantly lower overall and clinical pregnancy rates (45.4% vs. 73.3%) with respect to those in cycles with LH < 13 mIU/mL on the day of ovulation induction (36.4% vs. 65.9%). The authors suggested that, if natural ovulation process is already started (LH level ≥ 13 mIU/mL), hCG administration should be avoided, postponing the embryo transfer after spontaneous follicular rupture [17].

## 7. Maternal and Neonatal Outcomes 

The wide use of PGT to implement assisted reproduction results, raises a concern about the potential risks of embryo biopsy, and extended embryo culture [10,180,181]. TE biopsy removes cells that are destined to form the placenta, increasing the risk of pathological placentation, potentially responsible for pre-eclampsia and reduced fetal growth. Therefore, the comparison of adverse obstetric and neonatal outcomes in pregnancies obtained from PGT IVF cycles and traditional IVF is mandatory.

In the study by Scott et al. [185], only 30% of biopsied embryos had sustained implantation and developed into live-born infants, versus 50% of non-biopsied controls. In contrast, sustained implantation rates were equivalent (51% vs. 54%) for biopsied and control blastocysts.

Zhang et al. [176] aimed to evaluate the existence of a correlation between TE biopsy and blastocysts quality. The authors found that, in high quality blastocysts, there were no differences in the survival and implantation rates, independently of the number of cells removed. However, blastocysts with grades B and C had significantly lower implantation rate, with an increasing number of TE cells removed. Implantation potential is negatively affected by the number of TE cell removed for the analysis in blastocysts with poor morphological score.

Additionally, a reduced cumulative HCG secretion after the biopsy procedure was suggested by Dokras et al. [186]. Moreover, the authors evidenced that the removal of less than 10 cells reduce the HCG secretion (87.6 +/− 24.8 mIU/mL), but the difference was not significant. Oppositely, when a large biopsy was performed (greater than 10 cells), the HCG levels fell to 19.9 +/− 9.1 mIU/mL. This study indicates that blastocyst biopsy might impair the blastocysts development. 

Forman et al. [10] reported neonatal and obstetric outcomes, when comparing SET after blastocyst biopsy and untested double embryo transfer in a randomized controlled trial. The delivery rates were similar (69% vs. 72%) through the fresh cycle and up to one frozen transfer, with a dramatic difference in multiple births (1.6% vs. 47%). The risk of preterm delivery, low birth weight, and admission to neonatal intensive care were significantly higher after untested two-embryos transfer. The improved obstetrical and neonatal outcomes suggest that PGT of a single euploid embryo might be a valid approach to patients requiring IVF.

On the other hand, a large study examining maternal and neonatal outcomes after TE biopsy documented a statistically significant increase in the risk of preeclampsia (10.5% vs. 5.8%) and placenta previa (4.1% vs. 1.4%) among pregnancies after IVF with PGT compared to those from IVF without PGT. The incidence of gestational diabetes, preterm premature rupture of membranes, and postpartum hemorrhage were similar. In addition, no differences were noted between the groups regarding neonatal outcomes, such as gestational age at delivery, preterm birth, low birth weight, neonatal intensive care unit admission, neonatal morbidities, or birth defects. [187].

The evaluation of 1721 children born after IVF cycles, including PGT at blastocyst stage combined with FTET, only showed an increased rate of cesarean section. In singletons, the cesarean section rate was as high as 80%, reached more than 90% in twins and it was significantly increased in PGT group when compared with controls [188].

Jing et al. [189] evaluated obstetric and neonatal outcomes from pregnancies that were obtained from FTET after TE and cleavage-stage biopsy for PGT. The results demonstrated that the incidence of gestational hypertension was significantly higher after blastocyst biopsy, as compared with cleavage-stage embryo (9.0% vs. 2.3%). Birth-weight and gestational age were higher after blastocyst-stage embryo transfer when compared to cleavage-stage in twins, but no significant differences were detected in the incidence of perinatal deaths, birth defects, neonates gender, and birth-weight for gestational age in both singletons and twins.

## 8. Conclusions

The preimplantation genetic testing is a valid technique to evaluated embryo euploidy and mosaicism before transfer. Next generation sequencing is considered by several studies as the best molecular test and trophoectodermal biopsy at the blastocyst stage is today the most used method for embryo biopsy. Preimplantation genetic testing is currently under study for assessing its usefulness, safety, and clinical validity. The clinical application of PGT-A are mainly those conditions in which the risk of embryo aneuploidies might increase, such as advanced maternal age, recurrent pregnancy lost, repeated implantation failure, severe male infertility factor, or when a single embryo transfer is necessary. The clinical benefit of this strategy in good prognosis patients and egg donation programs should be assessed by properly designed randomized control trials, especially if single embryo transfer is requested. Maternal and neonatal outcomes seem to be reassuring but more studies are needed. Mosaic embryo should be considered for transfer after an appropriate genetic counseling for the transfer for patients without euploid embryos.

## Figures and Tables

**Table 1:** Clinical outcomes after transfer of mosaic embryos.

References	No. of Mosaic Blastocysts	Pregnancy Rate	Implantation Rate	Miscarriage Rate
[165]	18	33%	44%	11%
[157]	44	15.4%	30.1%	55.6%
[166]	143	41%	53%	24%
[155]	78	30%	38.5%	7.8%
[167]	100	30%	38%	7%

## Abbreviations

PGT	Preimplantation genetic testing
IVF	in-vitro fertilization
SET	Single embryo transfer
PGT-A	Preimplantation gentic testing for aneuploidies
LB	Live birth
FISH	Fluorescence in Situ Hybridization
aCGH	Array-Comparative Genomic Hybridization
NGS	Next Generation Sequencing
rtq-PCR	Real Time Quantitative Polymerase Chain Reaction
PB	Polar body
ICSI	Intracitoplasmatic sperm injection
TE	Trophoectoderm
BF	Blastocyst fluid
WGA	Whole Genome Amplification
AMH	Anti-Müllerian hormone
AMA	Advanced maternal age
FTET	Frozen.thawed embryo transfer
RPL	Recurrent pregnancy loss
PGT-M	Preimplantation gentic testing for monogenic disease
DOR	Diminished ovarian reserve
RIF	Repeated implantation failure
SMF	Severe male factor
KS	Klinefelter syndrome
WHO	World Health Organization
NOA	Nonobstructive azoospermia
DFI	DNA fragmentation index
SCOS	Sertoli cell-only syndrome
DE	Donor egg
